# Supplementary material for: Are carbon emissions trading and green financial instruments synergistic? -Comprehensive quantitative research based on content analysis
Source: PLoS One. 2024 Mar 7;19(3):e0298601. doi: 10.1371/journal.pone.0298601 (PMC10919601; doi:10.1371/journal.pone.0298601)
Supplement: S1 Data — (ZIP) [file pone.0298601.s001.zip › Data/地级市Z_F工作报告.zip!/╝¬┴╓/│ñ┤║╩╨/└┤╫╘╣1⁄2╓┌║┼í╛┬φ┐╦╩2╛▌═°í┐_2010.docx]

各位代表：

　　现在，我代表市人民政府，向大会作工作报告，请予审议，并请列席会议的同志提出意见。

　　一、2009年工作回顾

　　2009年，是长春在困难挑战中阔步前进的一年。在市委的正确领导下，在市人大、政协的监督和支持下，我们紧紧依靠和带领全市人民，成功扭转了2008年下半年以来经济持续下滑的被动局面，有效保持了经济社会平稳较快的发展态势，圆满完成了市十三届人大二次会议确定的各项任务。

　　预计地区生产总值完成2919亿元，增长15%，经济增长速度继续在全国15个副省级城市中保持前列。全口径财政收入完成450亿元，增长21%；规模以上工业实现产值4400亿元，增长22.2%；全社会固定资产投资完成2300亿元，增长31.9%，这三项指标三年增长了一倍以上，均提前两年完成了五年翻番的目标。

　　一年来，我们主要做了以下工作：

　　（一）壮大农村经济

　　新增30亿斤商品粮工程取得阶段性成果，实现了404万亩耕地测土配方施肥，完成了520万亩耕地深松整地，实施了800万亩耕地生物防螟，2.4万农户配备抗旱水具。人工增雨防雹作业系统完成升级改造。尽管先后遭受低温、伏旱、雹灾影响，粮食产量仍然稳定在160亿斤以上的阶段性水平。

　　农业机械装备水平大幅提高，大中型拖拉机超过3万台，两年增加10倍。机械化已覆盖除玉米收割以外的大部分农耕作业，正在催生农业农村生产方式新的变革。

　　启动建设7大牧业产业园区，新增500多个牧业小区；规划建设20个特色种植园区，新增经济作物面积6万亩，农业结构调整迈出新的步伐。

　　县域经济快速发展，完成工业投资325亿元。县域生产总值、财政收入、规模以上工业产值分别增长20.3%、21.5%和30%。农安县财政收入突破10亿元，成为我市首个财政收入超10亿元的县（市）。

　　德惠、农安、榆树进入省级卫生城市行列。绿化美化村屯598个，1130公里农村公路投入使用，5万余户农村泥草房完成改造，18.7万农村群众解决饮水安全问题，农村生产生活条件得到新的改善。

　　（二）加快工业发展

　　出台了支持工业企业的若干政策，调动各种积极因素促进工业增长。规模以上工业实现利润260亿元，增长38.3%。开发新产品510种，实现新产品产值1995亿元，新产品产值率达到45.3%。工业增长速度、质量同步提升，对全市经济形势全面好转起到决定性的作用。

　　为应对国际金融危机，春节以后迅速掀起外出招商热潮，先后开展重大招商活动12次、小规模团组招商700余次。这些招商活动坚定了国内外客商特别是央企投资长春的信心，年初确定的150个重大项目全部按计划顺利实施，完成工业投资1090亿元，产业发展呈现逆势而上的喜人态势。

　　80万辆整车、80万台发动机、55万台变速器项目同时在建，一汽、大众、马自达、丰田、通用世界汽车产业5大巨头，电装、江森、德尔福、麦格纳、采埃孚世界排名靠前的5大零部件企业均在长投资建厂。

　　投资20亿元、建筑面积26万平方米的轨道客车园一期工程竣工投产，1000辆高速动车组基本形成生产能力，未来3年的市场订单总额达到550亿元。

　　热电三厂、热电四厂、华能电厂并网发电，新增发电能力242万千瓦，接近现有能力的1倍。

　　大成100万吨玉米化工醇一期工程竣工，新增160万吨结晶糖生产能力，色氨酸等下游产品实现工业化生产。

　　总投资30亿元的中冶京诚项目，一期工程完成投资12亿元，当年开工、当年建成、当年试生产。

　　亚泰五线日产5000吨水泥熟料、新大100万吨炼油等一批项目形成生产能力，投资百亿元的兵装新能源等一批新的扩能项目正式奠基。

　　这些项目全部建成投产后，全市工业产出能力有望实现翻番式增长。长春工业已经站在了一个新的发展起点上。

　　（三）大力发展服务业

　　3家股份制银行挂牌营业，兰宝信息成功复牌，金融机构存、贷款余额分别为4520亿元、3894亿元，增长47.5%和36. 2%，金融服务能力得到实质性提升。

　　4家投资超10亿元的商务综合体开工建设，7家区域性批发市场建设顺利实施，一个服务全省、辐射周边的服务体系正在形成。

　　与国家农业部共同举办的农博会集中展示了中国农业60年辉煌成果，生物产业大会展现了中国最前沿的生物产业技术，东博会国内外政要巨贾云集、盛况空前，汽博会、民博会、雕塑展、消夏节、冰雪节、房交会、中日韩友城大会、东北四市市长峰会都办出了新的水平。

　　适时出台了活跃房地产市场的22条政策，二手房成交量增长23%，商品房交易量增长27%。日均240辆左右小汽车落籍，全年净增小汽车9万辆，小汽车正在快速进入普通百姓家庭。消费需求日益旺盛，全社会消费品零售总额实现1097亿元，增长16%。

　　制定出台了文化产业扶持政策，实施了知合国际动漫产业园、御龙温泉等60多个文化旅游项目，文化产业增加值增长31%，占GDP比重提高近1个百分点。

　　接待国内外游客2241万人次，同比增长16.8%。机场旅客吞吐量380万人次，增长24.6%。外地居民购买增量住房占比达到16.3%，提高4个百分点。域外需求已经成为拉动城市消费增长的重要力量。

　　（四）建设绿色宜居城市

　　去年，长春进行了前所未有的大拆迁、大建设，西南工业区开始向绕城高速以外摆放项目，长东北起步区基础设施框架基本形成，北部新城路网建设顺利推进，以西客站为核心的西部新城正式开工，南部新城核心区10平方公里基本完成“七通一平”，城市大发展的空间框架已经拉开，城市化进程已经进入一个新的阶段。

　　长松高速、机场航站楼二期工程、长春站综合交通换乘中心开工建设，轻轨三期、长吉城际铁路、哈大高铁分别完成主体工程量的45%、80%和90%，地铁一期前期工作进展顺利。

　　渠化改造路口31处，大中修道路46条，新建续建桥梁14座、道路137条。除7座公铁立交桥尚未竣工以外，全长67公里的四环路基本实现全线贯通。

　　实施了一大批市政公用基础设施项目，新增集中供热面积300万平方米，电网投资相当于前三年总和，燃气供应能力增加1倍以上。

　　高标准启动实施了机场路风景林带建设，新建改造60多块精品绿地，鲜花彩化70多条街路，新增城区绿地300多公顷。

　　三佳湖、国际汽车公园2个公园开工建设，朝阳、动植物、君子兰3个公园改造顺利推进，友谊、锦绣、岱山、御花园、共青团5个公园和儿童公园玫瑰园、长春公园野生花卉园2个特色园中园向游人开放。

　　石头口门水库建成12平方公里芦苇湿地，实施了生物治水新模式。新立城水库移交属地管理，水源保护治理工程全面启动。

　　南部污水处理厂投入运行，东南和4县（市）污水处理厂建设顺利推进，北郊污泥处置场、蘑菇沟垃圾场开工建设。单位生产总值综合能耗、化学需氧量、二氧化硫排放量均完成年度目标，空气质量优良级天数保持340天以上。

　　市区建设用地控制性详细规划实现全覆盖。新一轮土地利用规划大纲获得批准，新增城市建设用地482平方公里，可以满足未来10年发展需要。坚持节约集约利用土地，土地管理工作得到加强。

　　向城区下放市政设施维护等一批管理权限，坚持不懈地整治非法广告、马路市场、货车违法占道停车等城市顽疾，全天候保洁、冬季清雪长效机制更加完善。

　　（五）切实保障和改善民生

　　去年，我们实施了2009民生行动计划，年初确定的98件民生实事，除3件跨年工程外均按计划完成。各县（市）区、开发区也都结合各自实际，创造性地为群众办了一大批好事、实事。

　　城区城镇低保月补助标准提高60元，城区农村低保年补助标准提高200元，城区重度残疾人低保金上浮20%，低保边缘家庭重度残疾人开始享受每月100元救助，养老保险金月均标准提高80元，失业保险金月标准提高112元。城市居民年人均可支配收入增加1274元，农民年人均纯收入增加393元。

　　新增城镇就业11.8万人，城镇登记失业率3.75%。高校毕业生落户做到“零门槛”。728名低保特困家庭大学毕业生在政府帮助下实现就业。7.7万名下岗失业人员实现再就业。“零就业家庭”基本保持动态为零。

　　2300余名断保困难职工通过政府贴息贷款方式实现社保接续，3585名改制企业遗失档案职工补办养老保险手续，4728名未参保集体企业退休人员进入养老保险体系，7193名未参保改制企业退休人员进入医保。全年新增养老保险7万人、失业保险6万人、工伤保险6万人、生育保险15万人、医疗保险83.2万人。

　　深入开展“万户特困户结对救助”、“万台洗衣机送万家贫困众得到不同形式的资助。3000余名贫困群众得到法律援助。

　　贫困家庭84名聋儿、119名先天性心脏病患儿、197名唇腭裂患儿、855名有社会危害的重症精神病人得到医疗救助。3968名贫困白内障患者接受免费复明手术，基本上做到“有一助一”。为贫困重度下肢残疾人募集7840台轮椅，首批1800台已经免费配发到位。

　　制定出台了养老服务业扶持政策，建成100个老人社区日间照料站，为101家社会养老机构发放运营补贴，100余名患有精神疾病的农村五保老人实现集中托管治疗。依法取缔8家问题养老机构，改善了49家养老机构办院条件。

　　健康城市建设工作全面启动，健康行动计划扎实推进，8000余名群众接受体质检测和健身指导，4万余名已婚待孕女性进行优生筛查和出生缺陷干预，40万名群众接受重点疾病早期筛查和预防指导，越来越多的市民经常参加健身活动。长春进入全国首批人口计生综合改革示范市行列。

　　新建7所农村寄宿制学校，改善9所特殊教育学校办学条件，取消19所义务教育阶段改制校，完成132所中小学危旧校舍改造。14所城郊中小学配备班车。农村中小学远程信息化教育覆盖率达到100%。汽车高专移交地方管理，依托职业技术学校组建了7个职业教育集团，全年完成各类职业培训50万人次。

　　严厉打击格调低下的不良广告和网吧接纳未成年人行为，全面加强和改进未成年人思想道德建设，长春经验被中央文明办在全国推广。

　　送戏下基层100场，免费放映数字电影2.2万场，举办了200余场广场演出、722场专业艺术演出以及文化艺术周、农民文化节、欢乐庄稼院等一系列活动，群众文化生活日益活跃。

　　启动建设东北亚现代文化名城。创作编排6个精品剧目。申报保护了5个历史文化街区，公布了72处第八批市级文物保护单位。省图书馆奠基。省科技文化中心主体落成、开始布展。中国光学科技馆落户长春，我市拥有了首家国家级展览馆。

　　新建购买廉租房12.5万平方米、廉价房30万平方米，为8500户贫困家庭解决了住房问题。2.5万户居民享受到二手住房交易税费减免优惠。改造棚户区、危旧房248万平方米，一大批市民住房条件得到改善。

　　加快解决历史遗留问题，500余处二次供水设施完成接收改造，1万余户交费未开栓燃气用户开栓供气，7万户无籍房通过审核具备办理产权条件。

　　扎实开展安全建设年活动，累计投入资金6.52亿元，排查整改安全隐患2万多个，全市安全事故同比下降12.9％。

　　新建消防站14座，购买消防车40辆，消防水鹤基本实现城区全覆盖。近半数农村乡镇实现“一乡（镇）一车一站”的消防能力建设目标。

　　严厉打击酒后驾车、货车超速超载、车辆冒牌假牌行为。年内发生的50余起交通肇事致死逃逸案件全部告破。社会治安打、防、控能力明显提升，成功破获和处置“9.29”银行抢劫案、“12.29”劫持人质案等一批重特大案件，年内重大恶性案件“零积案”。坚持不懈地打击黄、赌、毒等丑恶现象，社会治安状况进一步好转。

　　科学有效应对甲型H1N1流感，疫情始终处于散发可控状态。2户有资质的企业生产1700万人份甲流疫苗，接近全国产量的1/6，长春为全国甲流防控工作做出了贡献。

　　（六）加强政府自身建设

　　自觉接受人大及其常委会的法律监督、工作监督和政协民主监督，坚持重大事项向人大报告、与政协协商制度。定期听取人大代表、政协委员和各民主党派、工商联以及无党派人士的意见和建议。4件人大议案、1件政协建议案、246件人大代表建议和325件政协提案办复。

　　主动邀请人大、政协、法院及政府内部监督部门面对面地开展批评监督，对政府工作瑕疵、行政败诉案件、审计违规问题逐项进行整改，并建立了标本兼治的长效机制，政府行政能力进一步提高。

　　充分发挥市长公开电话作用，畅通了政府与群众的联系。及时解决媒体反映的各种问题5000多个，领导干部通过读报掌握社情民意、弥补工作缺失、满足群众诉求的机制日渐完善。

　　实施新一轮政府机构改革，进一步理顺了部门职责关系，强化了公共服务职能。

　　深入开展学习实践科学发展观活动，解决了一批影响制约科学发展的体制机制问题和经济社会发展中的突出矛盾。

　　认真开展“认识新变化、推动新发展”研讨活动，深刻分析经济社会各方面的新变化，有针对性地调整工作思路和政策措施，努力使政府工作更加理性、自觉、富有成效。

　　坚持从严治政，廉政建设和反腐败工作取得新成效。启动了“万人评议机关”活动。取消、降低、暂停各类收费84项，减轻企业和社会负担上亿元。

　　深入推进信访积案化解年活动，解决了1200多个事关群众切身利益的实际问题。

　　国家安全、质监、统计、民族、宗教、外事、侨务、档案、保密、人防、地方志、红十字、妇女儿童、民兵和预备役等工作都取得了新成绩。

　　各位代表！一年来所取得的成绩，凝聚着全市各族人民的心血和汗水，体现着各方面的帮助和支持。在这里，我代表市人民政府，向为长春发展做出无私奉献、付出辛勤劳动的全市各族人民致以亲切的问候和崇高的敬意!向给予我们支持与监督的人大代表、政协委员，各民主党派、工商联和无党派人士、人民团体，向参与长春发展建设的中省直单位、驻长部队指战员、武警官兵和港澳台同胞、海外侨胞及国际友人表示衷心的感谢!

　　看到成绩的同时，我们也清醒地认识到，经济社会发展和政府工作中还存在不少问题：

　　——经济回升基础还不牢固，保持经济平稳较快增长的压力仍然很大。

　　——长期形成的经济结构性矛盾日益突出，加快转变发展方式成为当务之急。

　　——农业“靠天吃饭”的局面尚未根本改变，以节水抗旱为重点的农业基础设施建设需要进一步加强。

　　——出口总额、劳务输出人数持续下滑，机电、高新技术产品出口下降，新增外资企业数量减少。

　　——部分低收入群众生活还很困难，涉及群众切身利益的问题需要进一步解决，群众利益诉求反映渠道有时还不够畅通。

　　——城市承载能力有待进一步提高，冬季低温寒潮暴露了供热系统存在的问题，交通拥堵矛盾日趋突出。

　　——公共安全事故、灾害事故隐患较多，食品药品安全监管水平需要进一步提高。

　　——社会事业体制性、机制性和结构性矛盾有待于进一步解决，公共服务不够完善，还不能完全适应群众需求日益增长的新形势。

　　——政府职能转变不够，个别工作人员公仆意识不强，发展软环境有待于进一步改善，官僚主义和腐败现象仍然存在。

　　这些矛盾和问题，需要我们认真面对，下决心予以解决。

　　二、2010年主要任务

　　今年，是“十一五”规划的最后一年，也是长春老工业基地振兴的关键之年。为了把长春业已形成的大好形势保持下去，我们不仅要继续推动经济社会平稳较快发展，更要在促进发展方式转变上下功夫，在发展中促转变，在转变中求发展，努力使发展建立在结构优化、效益增加、后劲增强的基础之上。

　　今年政府工作的指导思想是：深入落实科学发展观，坚持统筹推进工业化、城镇化和农业现代化，牢牢把握加快发展、改善民生、建好城市、促进和谐“四大重点”，更加注重保持经济平稳较快增长和积累发展能力，更加注重优化经济结构和转变发展方式，更加注重改革开放、自主创新和发展民营经济，更加注重生态建设和城乡环境建设，更加注重改善民生和维护社会和谐稳定，把长春发展不断推向新的阶段。

　　全市经济社会发展主要预期目标是：地区生产总值增长15％，总量突破3000亿元；全口径财政收入同步增长，总量突破500亿元；全社会固定资产投资增长30％左右，总量接近3000亿元；规模以上工业产值增长20％以上，总量突破5000亿元。实现这样的目标，长春的经济实力将迈上一个新的台阶。

　　重点抓好以下八项工作：

　　（一）做大做强工业

　　滚动实施150个重大项目，完成1400亿元的工业投资，进一步提升工业产能、优化工业结构，推动工业在增量扩张过程中实现质的提升。

　　继续实施汽车“五个一”工程，新增百万辆整车、百万台发动机、百万台变速器、10万辆专用车、1000亿元零部件产值项目力争全部在建，努力建设世界级的汽车产业基地。

　　实施轨道客车产业园二期工程，1000辆城市轨道客车形成生产能力，启动机车厂搬迁改造，全力打造集制造、研发、维修、零部件生产于一体的世界级轨道客车产业基地。

　　启动建设100万吨玉米化工醇二期工程，化工醇正式投产，聚酯、树脂、化纤等下游项目建设取得实质性进展，努力建设世界级的玉米化工产业基地??双阳油气开发、亚泰水泥六线、中机北方二期等一批重大项目，加快发展建筑建材、石油天然气、农业机械等优势产业。

　　加大自主创新力度，抓好100个重大科技成果转化，扶持100户高新技术成长型企业。实施兵装新能源、混合动力汽车、激光及平板电视、光伏发电、基因工程等一批重点项目，发展壮大光电信息、生物医药、新能源、新材料四个高新技术产业。继续支持工业企业退城进开发区，推动传统产业优化升级。

　　加快建设装备制造业园、新能源产业园、光电子信息园、农机装备制造园和欧美、日系汽车零部件园等一批产业园区，努力形成一批有品牌、有规模、有影响力的产业集群。

　　发展工业，必须充分发挥开发区的主力军作用。新的一年，我们要继续支持开发区加快发展，在科技创新、环境保护、节约用地以及提高投入产出水平等方面实现新的突破。

　　（二）加强“三农”工作

　　推进新增30亿斤商品粮工程，改造18万亩中低产田，建设100万亩全程农机化示范区。实施3万亩旱田节水灌溉示范试点工程。深松整地500万亩，完成耕地三年深松一遍的目标。正常年景粮食产量力争达到180亿斤。建设10万套标准化新型农户储粮仓。新增经济作物种植面积10万亩。新建牧业小区400个以上。加强动植物重大疫病防控。

　　发展农业机械化，综合农机化水平提高5个百分点。培育各种形式的农机合作服务组织，加快建立农机社会化服务体系。

　　集中力量建设县域开发区和工业集中区，带动县域经济全面发展。四县（市）地区生产总值、全口径财政收入增长20%以上，规模以上工业产值增长30%左右。

　　高标准实施4个县城、16个重点镇的市政公用设施建设，发展社会事业，吸引更多农村人口向城镇集聚，使城镇化成为县域发展的新动力。

　　四县（市）都要争创国家级卫生城，各乡镇都要争创省级卫生乡镇，具备条件的村屯都要建设垃圾收集、沼气和自来水设施。

　　新建农村公路500公里，绿化美化村屯500个，改造农防林700公顷，解决15万农村群众饮水困难。

　　（三）全面提升中心城市服务功能

　　长春作为省会中心城市，聚集了全省优势资源。我们要努力建设服务全省的现代服务业体系，在服务全省的过程中发展壮大自己。

　　万达广场等4家商务综合体完成主体封闭，恒兴国际城等5家商务综合体开工建设。

　　农机现代物流科技园等10家市场建成开业，谋划启动一批专业化、特色化的区域性批发市场。

　　办好东博会、汽博会、农博会、房交会、电影节、冰雪节、消夏节、雕塑展和图书博览会等重大展会，不断提高会展规模和层次。

　　引进2家以上股份制银行、2家保险机构、3家风险投资机构，支持企业上市融资，启动城市金融区建设，进一步提升金融服务功能。

　　完善活跃房地产市场的政策措施，保持房地产业平稳健康发展。适度扩大土地供应规模，增加普通商品房供给，支持居民自住和改善性住房需求。

　　启动建设长影博物馆、奥林匹克中心、城市规划展览馆，省图书馆、汽车博物馆主体封闭，中国光学科技馆开始布展，省科技文化中心建成开放。加快发展动漫、创意、出版业，文化产业增加值占地区生产总值比重达到5％以上。

　　引进培育服务外包企业，建设服务外包产业基地，推动服务外包业成为新的经济增长点。

　　支持省内外企业在长设立地区总部、销售中心、结算中心和研发中心，争取更多的国内外知名中介机构落户长春。

　　整合发挥优势资源，进一步强化旅游、消费、教育、医疗、文化等方面的服务功能，吸引越来越多的人来长春旅游、购物、上学、就医、过周末。

　　（四）加快改革开放

　　23户进入破产程序的国有企业基本完成改制任务。妥善解决国企改革遗留问题。加强公益性企业国有资产监督管理。推进厂办大集体企业改革。

　　放宽市场准入，落实优惠政策，优化发展环境，发展壮大民营经济，主营业务收入增长20%以上。实施中小企业成长工程，建设创业孵化基地100万平方米，创办中小企业2000户以上。

　　完成规范改制校工作，依法规范义务教育阶段办学行为，积极支持民办教育，促进教育均衡发展。

　　深化医药卫生体制改革，推行国家基本药物制度，促进基本公共卫生服务均等化。

　　推进文化体制改革，市直剧院团、出版单位完成转企，广播电视基本实现制播分离。

　　深化农村土地制度改革，基本完成城区农村集体经济组织征地补偿费分配制度改革和集体林权制度主体改革。抓好农村土地承包经营权确权登记试点，有序推进土地承包经营权流转，引导家庭经营向生产集约化方向发展。继续开展城乡建设用地增减挂钩试点，加强土地节约集约利用。

　　抢抓长吉图上升为国家战略的有利契机，申报筹建长东北综合保税园区，扩大对内对外开放，启动综合配套改革，培育特色新兴产业，努力把长东北建设成为长吉图的起步区、新型产业的聚集区、综合配套改革的试验区。

　　开展招商引资九个月攻坚战，瞄准中央企业和国内外500强企业，引进一批战略性大项目。全年实际利用外资增长10%、利用内资增长15％。

　　出台扶持外贸出口的政策措施，扩大机电、高新技术产品出口，千方百计稳定出口形势，外贸出口增长10％以上。大力实施“走出去”战略，支持有条件的企业对外投资。加强外事侨务工作，进一步搭建对外合作交流平台。

　　（五）推进新型城市化

　　把扩大城市规模与消除城市病有机结合起来，走又好又快的新型城市化道路，努力建设绿色宜居城市。

　　加快建设“三城两区”。南部新城，启动人民大街南端中央商务区建设，实施净月长伊公路以西区域开发；西部新城，建设西客站场站及周边路网；北部新城，抓好北站房周边商业开发及路网改造；西南工业区，启动富锋分团开发；长东北，启动兴隆分团改造，建设绕城高速以内基础设施。

　　继续推进哈大高铁、长吉城际铁路建设。完成机场航站楼二期工程。主动配合长平高速扩建，长松高速建成通车，同步改造硅谷大街、亚泰大街北2个高速公路出入口。

　　地铁一号线力争开工建设，轻轨三期力争实现试通车，新建续建西客站、长春站2个综合交通换乘中心，初步形成城市快速轨道交通网络。

　　建设繁荣桥、公平桥，续建小南立交桥，畅通城市东西连接；实施远达大街连接线建设，增加1条南北大动脉；启动三环路分段拓宽改造工程，四环路全线建成通车。研究启动重要交通节点立体化改造，进一步提升城市通行能力。

　　改造城市电网，新增电网变电容量600兆伏安。建设燃气调峰环网，15万户居民煤气转换为天然气。

　　高标准实施长双公路和四环路绿化，绿化改造3个城市出入口，新建10个绿化精品、50处大块绿地，彩化美化100条主要街路。

　　新建续建同心湖、兴隆湖、光明、苗圃4个公园，建成三佳湖、国际汽车公园2个公园，完成长春公园、动植物园、伊通河境静园3个公园改造，建设郁金香园等一批特色园中园。

　　实施伊通河市区段全线综合治理，力争3年内使防洪工程达到200年一遇标准、城市污水管网完成改造升级、生态环境实现整体提升。

　　推广生态治水模式，建设长东北、净月、新立城、波罗湖、太平池、南部、石头口门七个生态湿地。

　　发展低碳经济和循环经济，抓好节能减排，削减4000吨二氧化硫排放量、5000吨化学需氧量，万元GDP能耗降低5.5%。

　　实施“公交优先”战略，投放新能源公交车100台，新建改造一批公交场站和公交候车亭，不断提高公共交通服务水平，方便市民出行。

　　加强城市交通管理，推行单行线、错峰上下班等交通优化措施，严惩公路“三超”、酒后驾车、肇事逃逸等严重交通违法行为。抓好地铁等大型基础设施的工程组织，提前制定交通综合解决方案，最大限度减小对城市交通的影响。

　　完善以区为主的城市管理体制，综合整治市容环境、户外广告、露天烧烤。拆除违法建筑，延长主要街路保洁时间，进一步完善冬季清雪机制，不断提升城市精细化管理水平。

　　（六）着力改善民生

　　围绕生存性、安全性、发展性三大民生需求，制定实施2010民生行动计划，集中力量办一批关系人民群众切身利益的实事，让人民群众得到更多实惠。

　　千方百计扩大就业，开发就业岗位12万个。推荐创业促就业项目1200个，发放小额贷款5000万元。更加注重解决困难群体就业问题，新增残疾人就业2000人，为1000名低保家庭高校毕业生提供公益性岗位，7万名下岗职工实现再就业，保持“零就业家庭”动态为零。

　　努力提高城乡居民收入。继续提高企业退休人员基本养老金发放标准、重点优抚对象抚恤和生活补助标准、孤儿和农村五保老人供养标准。完善强农惠农政策，努力拓宽农民增收渠道。完善机关企事业单位职工工资正常增长机制、农民工工资支付保障机制。城市居民人均可支配收入、农民人均纯收入分别增长10%和7%以上。

　　支持困难群体参加养老保险，解决职工参保历史遗留问题，完善新型农村社??，继续扩大养老保险覆盖面。

　　进一步完善社会大救助体系，出台低保边缘户采暖、住房、教育、就业专项救助政策。设立社区民情信息员，听民声、体民情、察民意，及时解决群众关心的热点难点问题。

　　为低保家庭儿童提供托幼补贴。为新入学贫困家庭大学生提供奖学金。建立自闭症儿童康复教育中心。实施三年提升计划，不断改善特殊教育学校办学条件。

　　新增城镇居民医疗保险10万人，新农合在乡农业人口实现全覆盖，人均财政补贴提高40元，报销比例分别提高5个百分点以上。

　　为6040名贫困重度下肢残疾人免费配发轮椅，基本上做到“有一助一”。从今年开始，贫困家庭的聋儿、唇腭裂患儿、先天性心脏病患儿、白内障患者、有社会危害的重症精神病人，要发现一例、救治一例。

　　继续整顿规范养老机构，新建100个社区老年日间照料站，两年内每个城区、开发区都要新增400张以上公办养老床位。落实优惠政策，鼓励社会力量投资新建养老机构。

　　推进殡葬体制改革，基本服务严格执行政府限价，杜绝垄断经营和诱导消费行为，采取有效措施切实减轻群众丧葬负担。建立城区低保群众基本殡葬服务免费制度。倡导厚养薄葬，树立文明节俭办丧事的新风尚。

　　全面加大棚户区改造力度，力争用3年时间基本完成主城区集中连片棚户区改造任务。改造农村泥草房、危房4万户。购买2.5万平方米廉租房，新建配建60万平方米保障房。基本解决交费未开栓燃气用户供气问题，200万平方米无籍房完成确权。充分发挥社区作用，为弃管小区居民提供基本物业服务。

　　实施“暖房子”工程，大力发展热电联供、区域性大锅炉房，加快改造小锅炉房和供热管网，建立供热保障和监管的长效机制，用三年时间全面提升城市供热能力。年内完成500个小锅炉房并网改造，新增集中供热面积550万平方米。

　　深入开展安全建设年活动，毫不松懈地排查整改安全隐患，坚决遏制重特大安全事故发生。加快改造高危燃气管网，推广加油（气）站阻隔防爆技术，加强防震减灾基础设施建设，提升城市安全保障能力。启动全民安全教育基地建设。

　　以更坚决的态度开展食品药品安全专项整治，实施全过程、全方位、全行业的质量安全管理，让人民群众吃得放心、用得安全。

　　抓好公安信息化建设，继续实施天网工程，构建现代化治安防控网络。落实社会治安打、防、控措施，加大一般案件侦破力度，从群众身边事入手改善治安状况。严厉打击严重暴力犯罪，努力做到黄赌毒必治、命案必破、黑恶势力必除、“两抢一盗”必打。长春绝不允许任何一种治安问题成风，绝不允许黑恶势力滋生做大。

　　（七）大力发展社会事业

　　努力办好人民满意的教育。加强教师队伍建设。积极推进办学条件标准化建设，城区义务教育阶段学校三年内全部达标。继续实施中小学校舍安全工程。加强农村初中寄宿制学校和农村中心小学建设。完成汽车高等专科学校一期建设。

　　推进健康城市建设，全面实施健康行动计划。继续开展高危致病因素干预、重点疾病早期筛查、健康知识教育和全民健身行动。深入实施“情牵新生命、优生优育促进工程”，不断提高孕前优生筛查率、出生缺陷干预率、新生儿疾病筛查率。60%村卫生所完成标准化建设，乡镇、社区卫生服务机构完成基础配套设施建设。全力防控甲型H1N1流感等传染病。

　　大力发展群众文化。举办广场文化活动200场，每村每月免费放映一场数字电影。开展社区艺术节、农民文化节、欢乐庄稼院和全民读书等各种文化活动。基本实现广播电视村村通。深入开展扫黄打非，严格禁止网吧接纳未成年人上网，净化青少年成长环境。

　　建设东北亚现代文化名城。加大历史文化街区、历史建筑维修和保护力度，力争国家重点文物保护单位实现零的突破。支持艺术家开展精品文艺创作，提高城市文化品位。

　　弘扬“宽容大气、自强不息”的城市精神，广泛开展群众性精神文明创建活动，力争早日进入全国文明城市行列。开展“五五”普法。推进基层民主自治，组织好村委会换届选举。抓好双拥共建，力争实现全国双拥模范城“七连冠”。

　　（八）加强政府自身建设

　　自觉接受市人大及其常委会的法律监督和工作监督，主动接受市政协民主监督，继续坚持重大事项向人大报告、与政协协商制度。认真听取民主党派、工商联、无党派人士和各人民团体意见。办好人大代表议案、建议、意见和政协提案。

　　坚持依法行政，自觉接受司法监督，支持监察、审计部门依法独立监督，主动接受新闻舆论和社会公众监督，及时纠正违法、不当行政行为，不断改进政府工作。

　　加强信访工作，妥善处理人民内部矛盾。大力推行重大改革措施、重大项目社会风险评估预警机制，从源头预防和减少社会矛盾。进一步办好市长公开电话。继续开展读报活动。

　　加快电子政务建设，完善新闻发言人制度，大力推进政务公开。深化行政审批制度改革，进一步减少行政许可数量、压缩办理时限。抓好清费减负，切实降低企业负担，努力营造良好经济发展软环境。

　　完善公众参与、专家论证和政府决策相结合的行政决策机制，不断提高决策科学化、民主化水平。抓好“十二五”规划及相关重大问题的研究工作。探索完善科学合理的政府绩效评估指标体系和评估机制。抓好经济调查和人口普查。

　　坚持从严治政、廉洁从政，深入开展“加强廉政、效能风险管理，健全部门内部控制机制”活动，强化对权力运行的监督和制约。

　　认真开展“解放思想找差距，振奋精神促发展”活动。进一步加大“三满意”机关、学习型机关创建力度。深入推进“查找改”活动，不断提高政府行政效能。加强公务员教育、管理和监督，建设一支忠于职守、勤勉敬业、求真务实、廉洁自律的公务员队伍。

　　各位代表，长春已经站在一个新的历史起点上。牢牢抓住一切有利机遇，努力把长春各项事业不断推向前进，是我们光荣而神圣的使命。让我们在市委的坚强领导下，扎实工作，锐意进取，为建设繁荣、和谐、开放、美丽的新长春而努力奋斗！
